# The circadian night depression of photosynthesis analyzed in a herb, *Pulmonaria vallarsae*. Day/night quantitative relationships

**DOI:** 10.1007/s11120-022-00956-1

**Published:** 2022-09-10

**Authors:** Paolo Pupillo, Francesca Sparla, Bruno A. Melandri, Paolo Trost

**Affiliations:** Department of Pharmacy and Biotechnology, University of Bologna Alma Mater, Via Irnerio 42, 40126 Bologna, Italy

**Keywords:** Circadian rhythm, Chlorophyll fluorescence, Light response curve, Photosynthesis, *Pulmonaria*, Shade plants, Water stress

## Abstract

Although many photosynthesis related processes are known to be controlled by the circadian system, consequent changes in photosynthetic activities are poorly understood. Photosynthesis was investigated during the daily cycle by chlorophyll fluorescence using a PAM fluorometer in *Pulmonaria vallarsae* subsp. *apennina*, an understory herb. A standard test consists of a light induction pretreatment followed by light response curve (LRC). Comparison of the major diagnostic parameters collected during day and night showed a nocturnal drop of photosynthetic responses, more evident in water-limited plants and consisting of: (i) strong reduction of flash-induced fluorescence peaks (FIP), maximum linear electron transport rate (J*max*, ETR_*EM*_) and effective PSII quantum yield (Φ_PSII_); (ii) strong enhancement of nonphotochemical quenching (*NPQ*) and (iii) little or no change in photochemical quenching *qP*, maximum quantum yield of linear electron transport (*Φ*), and shape of LRC (*θ*). A remarkable feature of day/night LRCs at moderate to high irradiance was their linear-parallel course in double-reciprocal plots. Photosynthesis was also monitored in plants subjected to 2–3 days of continuous darkness (“long night”). In such conditions, plants exhibited high but declining peaks of photosynthetic activity during subjective days and a low, constant value with elevated *NPQ* during subjective night tests. The photosynthetic parameters recorded in subjective days in artificial darkness resembled those under natural day conditions. On the basis of the evidence, we suggest a circadian component and a biochemical feedback inhibition to explain the night depression of photosynthesis in *P. vallarsae*.

**Supplementary Information:**

The online version contains supplementary material available at 10.1007/s11120-022-00956-1.

## Introduction

A decrease of photosynthesis potential during night hours, including a drop of photosynthetic activity under artificial illumination that reproduces daily conditions, is long known. This effect, here defined as the circadian night depression of photosynthesis, was assigned to the expanding domain of circadian events following early reports on rhythmic photosynthetic activity in plants (Pallas et al. 1974; Kerr et al. 1985; Kloppstech 1985). However, most studies were focussed on circadian stomatal movements (Martin and Meidner 1971; Holmes and Klein 1986) and it was generally agreed that the daily changes in photosynthetic activity depended on control of leaf intercellular pCO_2_ by stomata (Raschke 1975; Cornic and Briantais 1991). Therefore, the nature and very existence of biochemical oscillations of photosynthetic potential during a daily cycle and independent of light was in doubt, and to some extent remains so.

Plants are subject to a daily cycle of light and dark which has its own effects on metabolism regardless of the biological clock (Zaffagnini et al. 2019). These effects are largely explained in terms of reduction of protein disulfides during light-to-dark transitions and vice-versa, resulting in fine tuning of chloroplast enzyme activities and regulation of photosynthesis on multiple levels (Michelet et al. 2013; Yoshida et al. 2019). Electrons for disulfide reduction are provided either by the photosynthetic linear electron transport chain via thioredoxins *f/m*, or by the oxidative pentose phosphate pathway via NADPH thioredoxin reductase C (Cejudo et al. 2019; Zaffagnini et al. 2019). Thioredoxins are also involved in the ROS-dependent, dark oxidation of targets (Yochochi et al. 2021). The light/dark and circadian regulations of photosynthesis are distinct but strictly cooperating and overlapped under natural daily cycles, and influence each other with mechanisms that are not yet fully understood (Dodd et al. 2014).

Evidence has accumulated for a circadian control of many aspects of the photosynthesis process (Hennessey et al. 1993; Nakahira et al. 1998; Dodd et al. 2014). The use of transgenic luciferase as reporter enzyme (Millar et al. 1992; Matsuo et al. 2006; Philippou et al. 2019) revealed the night turnover of Cab polypeptides and other components of the photosystems under circadian control, with nuclear-encoded sigma factors as important mediators (Belbin et al. 2017). Delayed Chl fluorescence has been shown to oscillate according to a circadian rhythm in several plants (Gould et al. 2009). Moreover, the circadian clock underpins the synthesis of Rubisco small subunit, Rubisco activase and other photosynthetic enzymes (Martino-Catt and Ort 1992; Liu et al. 1996; Farré and Weise 2012) as well as components of the photorespiratory pathway (McClung et al. 2000), and drives post-translational modifications of chloroplast proteins (Booij-James et al. 2002; Seo and Mas 2014). It appears, therefore, that most stages of the photosynthesis process are under circadian control (Millar 2016). There are good reasons, therefore, to suspect that the night depression of photosynthesis investigated here is not merely a matter of stomata and may include a basic biochemical component (Hennessey et al. 1993), the more so as the circadian assimilatory response is independent of the stomatal cycle in arabidopsis mutants (Dodd et al. 2004).

Little information exists about circadian effects on photosynthesis of plants in nature (Resco de Dios et al. 2012; Millar 2016). Field research is made difficult by intricate relationships of the circadian clock with stomatal responses and the plant and soil water status (Flexas and Medrano 2002; Daszkowska-Golec and Szarejko 2013), as well as light, temperature and other environmental cues (Martino-Catt and Ort 1992; Jones et al. 1998). Moreover, plant sugars and photosynthetic products are known to modulate the circadian oscillator (Haydon et al. 2013; Frank et al. 2018); in particular, sucrose supports the robustness of circadian responses in light and darkness (Philippou et al. 2019). Metabolite effects can probably explain the extreme transiency of photosynthetic circadian responses in dark-kept plants (Hennessey et al. 1993; Nakahira et al. 1998; Booij-James et al. 2002).

In a previous paper (Recchia et al. 2017) we applied slow Chl fluorescence analysis using the sensitive Imaging PAM 2000 instrument to investigate inverse relationships between intensity of photosynthesis and length of the lifecycle in perennial understory herbs. By the same technique, we here present a fluorescence kinetic study of the night depression of photosynthesis and of prolonged darkness (“long night”) in one of these species, the lungwort *Pulmonaria vallarsae apennina*, a relatively shade tolerant herb of Italian uplands. This plant lends itself well to this type of experiments thanks to its long vegetation period and the large, enduring leaves that can undergo repeated PAM tests unharmed. The results are consistent with control of photosynthesis in this species by the circadian system under mild water stress, and suggest a biochemical limitation as the main factor responsible for the circadian night depression of photosynthesis.

### Materials and methods

#### Plant growth

The experiments were carried out on two dozens of plants of *Pulmonaria vallarsae* Kerner subsp. *apennina* Cecchi et Selvi (Italian lungwort, family Boraginaceae), a perennial understory herb, relatively shade tolerant and widely distributed on the Italian peninsula along Apennine reliefs (Cecchi 2015). The plants were collected in several hilly sites of the Bologna district in summer 2018 and grown in 25 cm diameter, 27 cm high pots on clay compost soil with watering every 3 days in a naturally lit greenhouse under partial shading at the Orto Botanico of the University of Bologna.

#### Chlorophyll fluorescence analysis and related experimental procedures

Chl *a* slow fluorescence was assayed with the Imaging PAM 2000 fluorometer (Walz Gmbh, Effeltrich, Germany) and associated PIM^R^ software via a dedicated computer as described (Recchia et al. 2017). One area of interest (3 mm diameter) was chosen on the lower (abaxial) surface of the leaf under study, which was held in place by a horizontal clip of 26 × 34 mm. PAM fluorometer tests were performed in laboratory or in greenhouse during late spring and summer months of four years (July 2018 to September 2021). Nighttime tests were carried out in darkness. For long experiments, plants were either transferred to a small laboratory greenhouse or to a nearby dark closet for the time required. Water was usually withheld for 2–3 days in advance of each trial and for its whole duration, since lavishly irrigated plants exhibit reduced night depression of photosynthesis (and reduced stomatal responses, Raschke 1975; Lawson et al. 2010).

Prior to each test, consisting of an activating light pretreatment followed by a light response curve (LRC) plants were held in the dark for 15 min. Minimum fluorescence in dark-adapted samples (*F°*_o_) was then obtained by applying measuring light of low frequency (1 Hz) and maximum fluorescence in dark-adapted samples (*F°*_m_) was obtained through an 800 ms blue saturation pulse of 2.4 mE m^−2^ s^−1^. The maximum quantum efficiency of PSII (*F*_v_/*F°*_m_) was constant in all samples. Absorptivity was determined by the instrument (Abs = 1−R/NIR) before each test.

**Fig. 1:**
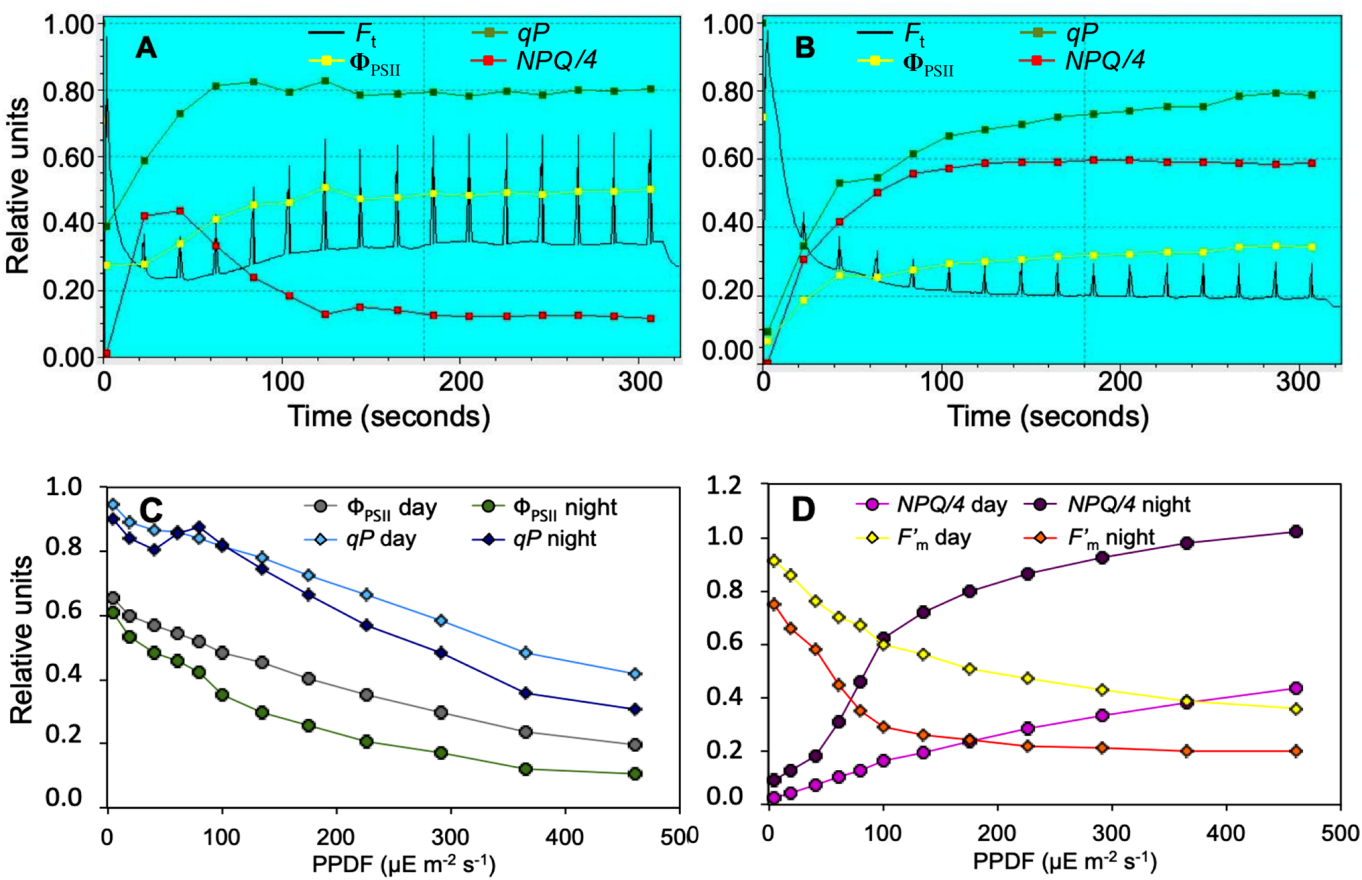
Activating light pretreatments (A, B) and subsequent LRCs (C, D) in a day/night experiment on a single *P. vallarsae* plant. **A**, morning test. Time-courses of *F*_t_ (with FIPs) continuous trace; Φ_PSII_ yellow dots; *qP* green dots; *NPQ* red dots. **B**, the same leaf tested at evening. **C**, LRCs: kinetics of parameters *qP* (cyano and blue dots) and Φ_PSII_ (green and grey); **D**, concomitant changes of *F’*_m_ (yellow and orange) and *NPQ* (violet and pink). All values relative to *F*°_m_ = 1.0, except that *NPQ* is divided by 4

The light pretreatment was conducted at actinic light of 100 µE m^−2^ s^−1^ with saturation pulses of 2.4 mE m^−2^ s^−1^ (duration 800 ms each) applied at 20 s intervals. This triggered 16 fluorescence peaks (FIP = *F’*_m_ – *F*_t_) of variable height, i.e. ~ 4% to 42% of *F°*_m_ depending on the state of the plant (see Fig. 1). Accordingly, FIPs are used in this work as a probe of PSII activity. Other fluorescence parameters were derived as follows: the effective quantum yield of PSII photochemistry is Φ_PSII_ = (*F’*_m_ – *F*_t_)/*F’*_m_; nonphotochemical quenching *NPQ* = (*F°*_m_−*F’*_m_)/*F’*_m_; photochemical quenching *qP* = (*F’*_m_ – *F*_t_)/(*F’*_m_ – *F’*_o_); photosynthetic electron transport rate ETR = Φ_PSII_ x PPFD × 0.5 x Abs (Björkman and Demmig 1987).

LRCs of electron transport rates (ETRs) were determined right after light pretreatments. LRCs were conducted at increasing steps of actinic light (from 1 to 460 μE m^−2^ s^−1^). The duration of each step was 20 s and ended with a saturation pulse of 2.4 mE m^−2^ s^−1^. Light fluorescence related parameters Φ_PSII_, *qP*, *NPQ* and ETR were calculated as above by PAM fluorometer software.

In day/night experiments plants were usually assayed around 08.30 am and 11.20 pm, and in “long night” experiments plants were assayed every 12 h. The total number of experiments performed was 252 with about 1300 single standard tests, but only tests with mature, healthy leaves having daily ETR_*EM*_ ≥ 20 µmol m^−2^ s^−1^ in the range of temperatures 19–28 °C were considered for the purpose of this paper.

#### Data processing

LRCs were analyzed by nonlinear regression (CoHort software) using the function of the Farquhar-von Caemmerer-Berry (FvCB) model (Farquhar et al. 1980; Farquhar and Wong 1984) yielding parameters J*max*, *Φ* and *θ*. In the FvCB model, J*max* is the calculated rate of linear electron transport under saturating irradiance, *Φ* is the maximum quantum yield of the linear electron transport and *θ* is a curvature factor. LRCs were also represented in double-reciprocal plots with linear responses at moderate to high irradiance (PPFD > 100 µE m^−2^ s^−1^), corresponding to rectangular hyperbolas in primary plots. When fitted to straight lines, LRC data give an estimate of ETR_*EM*_ (extrapolated maximum ETR for the high irradiance region of an LRC) and K_*PAR*_ (the PPFD at which ETR = ETR_*EM*_/2). ETR_*EM*_ approximated J*max* with deviations within + 15% of J*max* values. Other fluorescence and photosynthetic parameters are provided by fluorometer-associated PIM software.

## Results

Photosynthetic activity by day and night was investigated in leaves of potted, water-limited plants of *Pulmonaria vallarsae* on the basis of slow Chl fluorescence responses, recorded and elaborated by the Imaging PAM fluorometer. A standard test consisted of an activating light pretreatment followed by an LRC, derived from the fluorescence induced by a series of light steps of increasing energy.

Two standard tests conducted on the same *P. vallarsae* leaf by day and night are shown in Fig. 1 as a representative example. The same type of experiments was repeated on many plants, and the average values of fluorescence related parameters recorded at the end of the activating light pretreatments are listed in Table 1. In the morning (Fig. 1A), the leaf illuminated by actinic light of 100 µE m^−2^ s^−1^, responded to pulses of saturating light with a series of FIPs which soon reached their maximum height at 34% of *F*°_m_ (average value 26.2%; see Table 1). Simultaneously, *qP* and Φ_PSII_ reached steady state values, current fluorescence *F*_t_ rapidly dropped and partially recovered, and the dissipation parameter *NPQ* made a jump followed by stabilization at a low level (Fig. 1A).

**Table 1:** Means ± SD (*n* = 73) of parameters *F*_t_, FIP, *NPQ*, *qP*, Φ_PSII_ (A) and parameters J*max*, *Φ, θ*, ETR_*EM*_, K_*PAR*_ (B) for day/night PAM tests performed with *P. vallarsae* plants under natural light/dark illumination. Group A data are final values of the activating light pretreatment, group B data are calculated from the LRCs. Units as in Figs. 1–2, but *Φ* and *θ* are calculated by FvCB equation

	Day	Night	Day/Night ratio
A) Activating light pretreatment			
* F*_t_	0.33 ± 0.03	0.22 ± 0.03	1.50
FIP	0.26 ± 0.04	0.10 ± 0.01	2.58
* NPQ*	0.69 ± 0.13	2.08 ± 0.31	0.33
Φ_PSII_	0.44 ± 0.03	0.32 ± 0.03	1.38
* qP*	0.72 ± 0.06	0.69 ± 0.08	1.04
B) LRC			
J*max*	23.1 ± 1.2	13.2 ± 1.2	1.75
* Φ*	0.155 ± 0.011	0.144 ± 0.015	1.07
* θ*	0.76 ± 0.13	0.70 ± 0.15	1.09
ETR_*EM*_	26.8 ± 1.8	15.1 ± 1.2	1.77
K_*PAR*_	106 ± 13	50 ± 10	2.11

A quite different pattern was observed when the light induction pretreatment was administered during the night (Fig. 1B and Table 1). The most conspicuous difference consisted in small FIPs (10.1% of *F*°_m_ on average; see Table 1) which remained constant or nearly so during the whole pretreatment (low-photosynthesis state). At the same time there was a rapid rise of *NPQ* to an elevated, stable plateau symmetrical to a large drop of *F*_t_, while Φ_PSII_ increased to its maximum which was significantly lower than during day. Photochemical quenching *qP* rose with slow kinetics to the same final value as in the morning (Fig. 1B). In sum, at the end of the light activating pretreatment, the sample measured during the day was characterized by higher values of FIPs (2.6-fold) and Φ_PSII_ (1.4-fold) with respect to the night, while the sample measured during the night was typified by a higher level of *NPQ* (3.0-fold). On the other hand, the *F*_v_/*F*°_m_ parameter commonly used to characterize stress subjected plants did not vary at all between day and night (not shown).

The pretreatments were followed by LRC tests at increasing light intensities. Trends of Chl fluorescence related parameters as a function of PPFD are reported in Fig. 1C-D. During the day, the slow descent of *F’*_m_ with increasing irradiance was symmetrical to slowly rising *NPQ*, while Φ_PSII_ and *qP* declined linearly. In the night test, most parameters were significantly lowered in sharp contrast to a quick, strong rise of *NPQ*. *F’*_m_ underwent an abrupt biphasic fall, and the declining *qP* trajectory showed a distinct night hump around 80–100 µE m^−2^ s^−1^ PPFD.

**Fig. 2:**
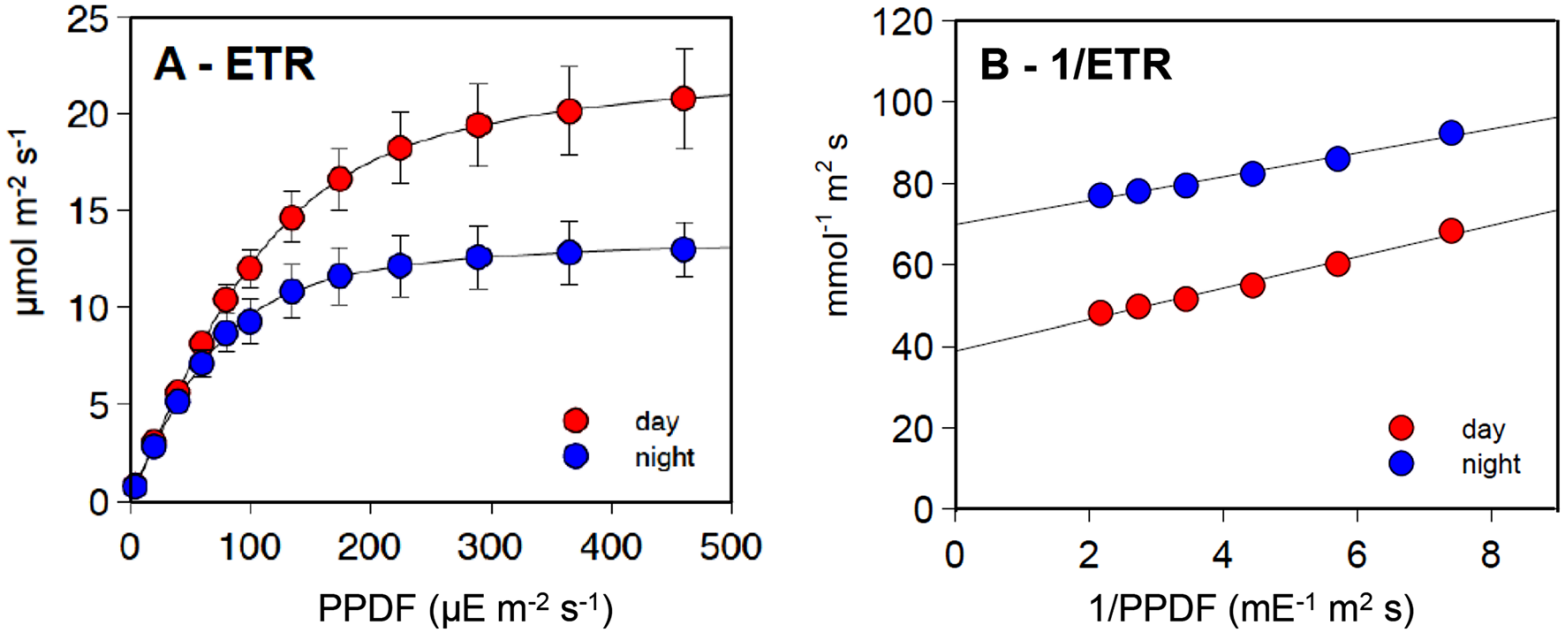
**A,** average ETR-LRC resulting from PAM fluorometry tests conducted between 2018 and 2021 (*n* = 73). Data are reported as weighed means ± SD and fitted with FvCB equation. **B,** linear interpolation of double-reciprocal plots of the same data (80–460 PPFD)

For both day and night tests, ETRs approximated a linear dependence on PPFD at low light intensities and an apparent hyperbolic response at higher light intensities (Fig. 2A). The linear responses in the low light range were very similar in day and night, while at high light intensities the hyperbolic responses diverged, with the day curve reaching much higher saturation levels. ETR data of each test were interpolated with the FvCB equation (Fig. 2A) allowing calculation of the relative parameters (Table 1). Mean LRCs for 73 day/night twin experiments are graphically summarized in Fig. 2A, while Fig. S1 reports single plant responses, with small differences between individual plants. The maximal ETR estimated as J*max* was on average 1.75-fold higher in day tests with respect to night tests, whereas the variations of the maximum quantum yield *Φ* and the curvature factor *θ* were very small (1.07 and 1.09, respectively). ETR data at moderate to high light intensitites (PPFD > 100 µE m^−2^ s^−1^) could be interpolated by straight lines in double-reciprocal plots (Fig. 2B) with extrapolated maximum values defined as ETR_*EM*_ having an overall day/night ratio of 1.77, similar to J*max.* Like J*max* and ETR_*EM*_, the half-saturation parameter of the LRC (K_*PAR*_) was also higher during the day (Table 1). The near parallelism of double-reciprocal plots implies that ETR_*EM*_ and K_*PAR*_ tend to keep a similar ratio of the respective day/night values.

**Fig. 3:**
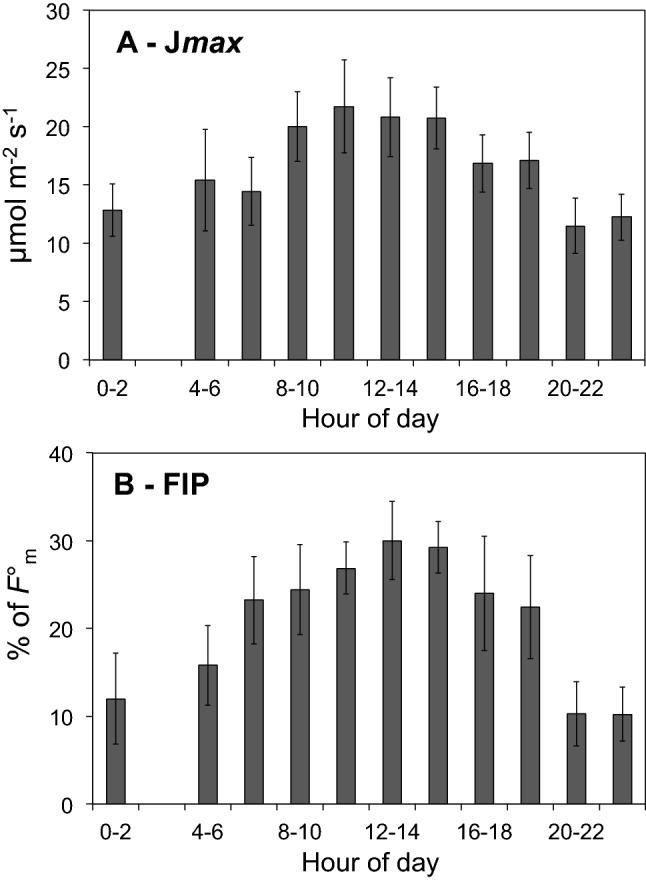
Daily courses of parameters J*max* **A** and FIP **B** under natural light–dark cycle. Data of tests conducted in summer months of years 2018–2020, normalized to 05.20 am sunrise corresponding to July 20th in Bologna (*n* = 141). Each bar represents mean ± SD of 6 to 32 pooled points over 2 h

The daily trends of photosynthetic parameters were examined on the basis of 141 tests carried out at different times of the day, normalized for 05.30 am sunrise and represented at 2 h intervals in Fig. 3. Maximum rates of electron transport (J*max*) showed a broad diurnal, almost symmetrical peak at 12–14 pm (Fig. 3A). The nocturnal value of J*max* was low and nearly constant between 9 pm and about 4.30 am and was followed by a sudden rise around dawn, indicating a very early onset of recovery of photosynthetic potential. This was confirmed by tests conducted before and after dawn on the same leaf in the absence of light (not shown). The FIP profile featured a sudden increase around sunrise with a sluggish ramp around 12–15 pm, and a quick drop in late afternoon and night (Fig. 3B), when *NPQ* prevails (Fig. 1D).

**Fig. 4:**
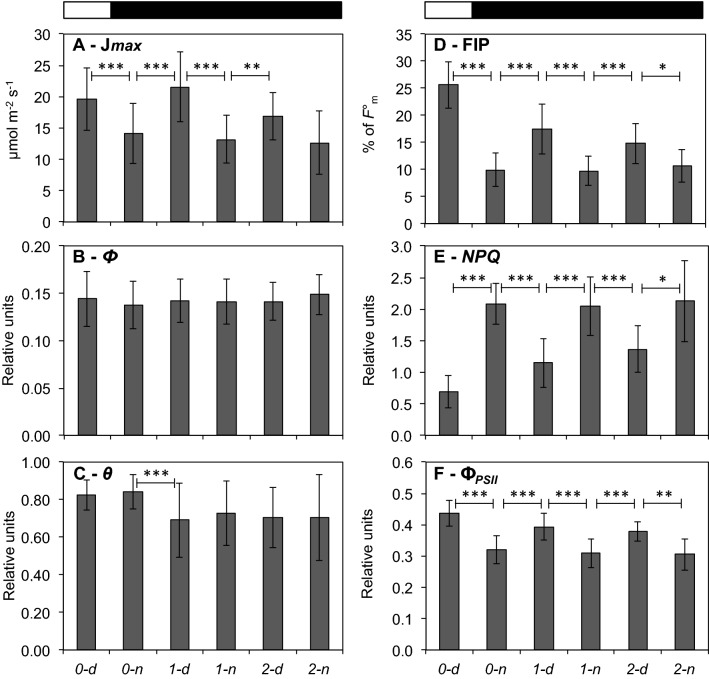
Average results of “long night” experiments for LRC parameters J*max* **A**, *Φ* **B**, *θ* **C** of the FvCB function, and fluorescence related parameters FIP **D**, *NPQ* **E** and Φ_PSII_ **F**, means ± SD (*n* = 36). Plants under natural light–dark regime were tested at ~ 11 am (*0-d*) and ~ 11 pm (*0-n*) of day 0, then transferred to constant darkness for 2 days (top black bar) and tested each day at 11 am (*1-d*, *2-d*) and 11 pm (*1-n, 2-n*) in the dark. Two-tailed t-test was used for statistica: *** *p* < 0.0001; ** *p* < 0.001; * *p* < 0.05

The pre-dawn enhancement of activity observed in several experiments suggested that changes of photosynthetic potential can occur in the absence of light in *P. vallarsae*, so the effects of prolonged darkness were probed (“long night” trials). Plants grown under natural light–dark cycles were tested at day 0 at morning (*0-d*) and evening (*0-n*), then transferred to constant darkness and assayed in the dark every 12 h at each subjective morning and night, and finally brought back to the light once the morning J*max* value had dropped to nocturnal levels. Pooled results of 36 experiments, each lasting 48–72 h, are summarized in Fig. 4, which documents the oscillatory trajectory of most parameters during a “long night” and the transiency of the effect.

In detail, FvCB interpolation of the ETR data shows that the maximum electron transport rate (J*max*) oscillates with circadian rhythmicity, with statistically supported day/night differences (Fig. 4 and Fig. S2). In the first subjective day in darkness (*1-d*), the J*max* value (like ETR_*EM*_) was fully recovered to the level of the previous morning in the light (*0-d*). The effect was attenuated during the second subjective day (*2-d*) but still visible if compared to night values, that were relatively constant over the whole experiment. No circadian rhythmicity affected parameters *Φ* and *θ*, even though the latter was significantly lower during the whole “long night” with respect to the usual light/dark conditions (Fig. 4C) suggesting a light-dependent component of *θ*. The fluorescence peaks FIPs, too, while exhibiting a robust rhythm were 24% lower (at *1-d*) during the “long night” than under normal light regime (*0-d*, Fig. 4D). In fact FIP, and Φ_PSII_ to a lesser extent, oscillated in phase with J*max* showing low values during the nights and attenuation of circadian oscillation towards the end of the experiments. By contrast, *qP* showed only small, insignificant oscillations (not shown). *NPQ* behaved exactly symmetrical to J*max*, FIP and Φ_PSII_ with opposite phasing; it was very low in the light (at *0-d*) and high and oscillating in subsequent darkness*.* Despite rapid damping, these results indicate that most photosynthesis and fluorescence parameters of darkened *P. vallarsae* leaves undergo oscillations of different strength between subjective days and subjective nights. Data within and between experiments also evidence a remarkable stability of J*max*, FIP and Φ_PSII_ in the night and, conversely, of *NPQ* in the day (Fig. 4). Interestingly, the general features of the photosynthetic responses recorded for J*max*, FIP, Φ_PSII_ and *NPQ* in subjective days and subjective nights were similar to responses in a normal light/dark cycle (compare Table 1 and Fig. 4).

**Fig. 5:**
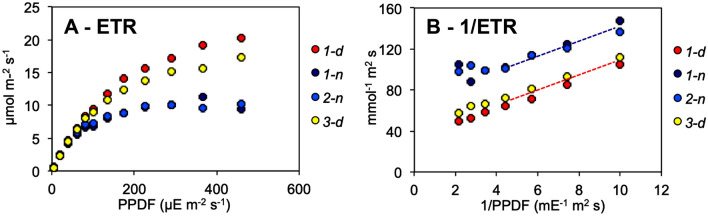
LRCs from a “long night” trial. **A**, a plant growing under natural light–dark regime was transferred at 08.00 pm of day 0 to constant darkness for 63 h and assayed every 12 h in darkness (see Fig. 4), then readmitted to light and watered, with a final test after 4 h light exposure (*3-d*). Four LRCs (*1-d*, *1-n*, *2-n*, *3-d*) out of seven are shown for clarity. **B**, same data treated as double-reciprocal plots in the high light range (100–460 µE m^−2^ s^−1^ PPFD)

Finally, an illustrative “long night” experiment is reported in Fig. 5A to highlight the oscillatory trends of photosynthetic electron transport, showing three selected LRCs in constant darkness (stages *1-d*, *1-n*, *2-n*) and the final LRC after a brief recovery of the plant in the light (*3-d*), all borne by the same leaf. Double-reciprocal plots (Fig. 5B) display straight/parallel patterns of the LRCs arranged in two distinct bundles for subjective day (high-photosynthesis state) and night (low-photosynthesis state). In fact, the ratios of subjective morning ETR_*EM*_ and K_*PAR*_ values to the respective nighttime values were almost identical in this and similar experiments, both parameters giving a day/night ratio of 2.2 for the first day in darkness (*1-d/1-n)*. This also confirms the autonomy of most circadian photosynthesis responses from the presence of light during the first 1–2 days of darkness.

### Discussion

Daily changes of photosynthetic activity were investigated by Chl fluorescence analysis using the Imaging PAM 2000 fluorometer in *Pulmonaria vallarsae* subsp. *apennina*, a perennial understory herb (lungwort) of Italian mountains. Thanks to the noninvasive PAM technique, changes of photosynthesis and fluorescence linked parameters of a single leaf could be followed for quite a time with repeated tests. Under natural light/dark cycles, LRCs taken by night in slightly water-stressed plants showed a strong depression of photosynthesis with respect to daytime values, somewhat recalling the response of heat exposed leaves (Sukhova et al. 2018). The night effect was smaller or even absent in fully watered plants, consequently only slightly water-stressed plants were used for the present experiments. In particular, night LRCs showed reduced electron transport rates at medium to high irradiances and little change at low irradiances. In terms of photosynthetic parameters this effect resulted in low nocturnal values of electron transport (J*max* or ETR_*EM*_), but constant or very small changes in maximum quantum yield (*Φ*), shape of the curve (*θ*) and photochemical quenching *qP*. Nonphotochemical quenching *NPQ* was strongly enhanced in night tests, thus probably contributing to a strong decrease of fluorescence (FIP, *F*_t_) and a moderate decrease of effective PSII quantum yield (Φ_PSII_).

There is a plethora of biochemical impediments with potential damping effects on photosynthetic responses during night. The night blockade of Rubisco by active site decarbamylation and tight binding of inhibitory sugar phosphates (Taylor et al. 2022), and of Calvin-Benson cycle enzymes through redox post-translational modifications and formation of inactive complexes (Michelet et al. 2013; Gurrieri et al. 2021; Zimmer et al. 2021) might curtail the supply of substrates and acceptors for the linear electron transport and ATP synthase, the latter being itself dark-inactivated in an oxidizing cellular milieu (Tezara et al. 2002; Hisabori et al. 2013; Vaseghi et al. 2018; Hashida et al. 2018). This, in turn, might cause a nighttime feedback inhibition of PSII activity and photosynthetic potential, with an enhanced high energy state of thylakoids when illuminated, and consequent induction of energy dissipation mechanisms at the level of PSII antenna complexes (Laisk et al. 1992; Bassi and Dall’Osto 2021). This sequence of events is consistent with the high *NPQ,* low fluorescence responses and decreased ETR and Φ_PSII_. It is also consistent with the invariancy of the maximum quantum yield (*Φ*) of linear electron transport at low light intensities, with minimized *NPQ* and no metabolic control. It seems likely, therefore, that the night drop of most photosynthetic responses results from a persistent decrease of some key metabolite(s) and/or inactivation of ATP synthase and Calvin-Benson cycle enzymes, including Rubisco. It is also possible that a CO_2_ limitation of Rubisco activity due to stomata closure in the nighttime would reinforce this cascade of events.

Morning photosynthetic recovery in *P. vallarsae* does not depend on light in the short run and can be reiterated in constant darkness using an artificial “long night” of 2(-3) days, only interrupted by short tests every 12 h. Under these conditions, the photosynthetic activity during subjective days (i.e. in artificially darkened mornings) had the same features as the photosynthetic activity observed in naturally illuminated mornings: high levels of J*max*, Φ_PSII_ and FIP, and low *NPQ*. Likewise, photosynthesis during subjective nights was depressed as under a normal light/dark cycle. The oscillatory changes of photosynthetic potential during a 52–72 h “long night” may be described as a daytime boost of photosynthetic activity (damping from the second day) over a low and relatively stable nighttime level of activity. This evidence suggests timing of photosynthetic activity by a circadian oscillator with an oncoming light requirement. The latter is presumably of metabolic origin since sugars and photosynthates are known to sustain the circadian rhythm (Haydon et al. 2013; Frank et al. 2018; Philippou et al. 2019).

A circadian oscillation of photosynthetic potential was sporadically described in different plants including green algae with different measuring techniques (Pallas 1974; Lonergan 1981; Fredeen et al. 1991; Hennessey and Field 1991; Dodd 2014; Noordally and Millar 2015). Stomatal movements too are regulated by the circadian system (Martin and Meidner 1971; Holmes and Klein 1986) and there is little doubt that the present biochemical events are accompanied, to some extent, by stomatal responses. Circadian rhythms of stomatal movement, hydraulic conductance and growth are enhanced by drought (Caldeira et al. 2014), and we also observed that water abundant lungworts underwent a smaller drop of night photosynthetic potential, or none, compared to mildly water-stressed plants that had been grown under sparing irrigation. It thus appears that water stress enhances a number of plant circadian functions more or less related to photosynthesis. Early observations of circadian regulation of photosynthetic potential under constant levels of internal CO_2_ (Hennessey and Field 1991; Fredeen et al. 1991) also supported the view that variable stomata conductance could not be the only determinant of the night depression of photosynthesis (Farré and Weise 2012). Nocturnal stomatal opening with high gas conductance is correlated with elevated photosynthesis performances and growth rates in several species, and has likely a genetic background (Caird et al. 2007; Resco de Dios et al. 2016).

The circadian nature of the night depression of photosynthesis is confirmed by the recovery process initiating at dawn with or without light, reminiscent of reports on early synthesis of antenna (Kloppstech 1985; Millar et al. 1992) and other plastid proteins (Dodd et al. 2014). A circadian rhythm of delayed Chl fluorescence under constant light has been recorded in arabidopsis and other species (Gould et al. 2009), also suggesting that the night depression effect involves photosynthetic functions besides stomata (see Dodd et al. 2004; Lawson et al. 2010). The expression of thioredoxins *f* and *m* mediating the light-dependent activation of Calvin-Benson cycle enzymes and ATP synthase is under circadian control (de Dios Barajas-López et al. 2011). Reactive oxygen species (Lai et al. 2012) and the redox state of 2-cys peroxiredoxins (Edgar et al. 2012), both potentially implied in the dark oxidation of redox-regulated proteins (Vaseghi et al. 2018), do also oscillate in arabidopsis leaves with circadian rhythm.

Circadian regulation of photosynthesis is mainly suggested in this paper by the recovery of a high photosynthesis state in subjective days in the absence of light. In fact, the high photosynthesis state established in either subjective or real days are similar and might in principle be achieved through analogous mechanisms. The light activation of photosynthetic metabolism is largely explained in terms of thioredoxin-mediated reduction of Calvin-Benson cycle enzymes (and related regulatory proteins Rubisco activase, CA1P phosphatase, CP12) and ATP synthase (Portis et al. 2008; Michelet et al. 2013; Hisabori et al. 2013; Gurrieri et al. 2021). In chloroplasts, thioredoxins and thioredoxin-regulated proteins are photosynthetically reduced by PSI through ferredoxin: thioredoxin reductase, or nonphotosynthetically by NADPH thioredoxin reductase C using NADPH derived from the oxidative pentose phosphate pathway (Zaffagnini et al. 2019). Light independent activation of redox-regulated chloroplast proteins is therefore possible, as also suggested by the high sensitivity of NTRC knockout mutants to prolonged darkness (Cejudo et al. 2019). The light-dependent and clock dependent regulation of photosynthesis do clearly interact, but whether they share a common mechanism of action still needs to be thoroughly investigated.

## Supplementary Information

Below is the link to the electronic supplementary material.

Supplementary file1 (DOCX 314 kb)

## Abbreviations

Abs: Absorptivity
Chl: Chlorophyll
ETR: Photosynthetic electron transport rate
ETR_*EM*_: Maximum extrapolated value of ETR
FIP: Flash-induced fluorescence peak
F°m: Maximum Chl fluorescence in dark-adapted plants
F’m: Maximum relative fluorescence under illumination
F°o: Minimum Chl fluorescence in dark-adapted plants
Ft: Current Chl fluorescence
Fv: Variable fluorescence
FvCB: Equation of Farquhar et al. 1980
*Φ*: Quantum yield of photosynthesis (Farquhar et al. 1980)
Φ_PSII_: Effective quantum yield of PSII photochemistry
J*max*: Maximum extrapolated rate of LRC (Farquhar et al. 1980)
K_*PAR*_: Half-saturation PAR for an LRC
LRC: Light response curve
*NPQ*: Nonphotochemical quenching
PAM: Pulse amplitude modulated (fluorometer)
PAR: Photosynthetically active radiation
PPDF: Photosynthetic photon density flux
PSII: Photosystem II
*qP*: Photochemical quenching
ROS: Reactive oxygen species
SD: Standard deviation
*θ*: LRC smoothing factor (Farquhar et al. 1980)
