# Supplementary material for: The circadian night depression of photosynthesis analyzed in a herb, Pulmonaria vallarsae. Day/night quantitative relationships
Source: Photosynth Res. 2022 Sep 10;154(2):143–53. doi: 10.1007/s11120-022-00956-1 (PMC9630222; doi:10.1007/s11120-022-00956-1)
Supplement: Supplementary file 1 — Supplementary file1 (DOCX 314 kb) [file 11120_2022_956_MOESM1_ESM.docx]

**Journal name:** [Photosynthesis Research](https://www.springer.com/journal/11120/)

**The circadian night depression of photosynthesis analyzed in a herb, *Pulmonaria vallarsae*. Day/night quantitative relationships.**

**Paolo Pupillo^1^, Francesca Sparla^1^, Bruno A. Melandri, Paolo Trost^1^**

^1^University of Bologna Alma Mater, Department of Pharmacy and Biotechnology, Via Irnerio 42, 40126-Bologna, Italy

e-mail address of the corresponding author: francesca.sparla@unibo.it

**Fig. S1.** Average ETR-LRCs obtained by PAM fluorometry from 73 twin day/night experiments using 11 different plants (see Table 1). Plant code (e.g. FAB) and number of tests for each plant are indicated. Morning tests red dots, night tests blue dots. Data are fitted with FvCB equation and reported as means ± SD.

**Fig. S2.** Average ETR-LRCs of the “long night” experiments of Fig. 4. Tests were performed in the first morning of each experiment in the light (*0-d*) and following two mornings in darkness (*1-d*, *2-d*), and in the corresponding nights (*0-n*, *1-n*, *2-n*). Each dot represents the mean of original data, curves are fitted with FvCB equation (n = 36).
